# Enhancing team success in the neonatal intensive care unit: challenges and opportunities for fluid teams

**DOI:** 10.3389/fpsyg.2023.1284606

**Published:** 2023-11-07

**Authors:** Elizabeth A. Bell, Gabrielle A. Rufrano, Allison M. Traylor, Bryan L. Ohning, Eduardo Salas

**Affiliations:** ^1^Clemson University, Clemson, SC, United States; ^2^School of Medicine Greenville, University of South Carolina, Greenville, SC, United States; ^3^Rice University, Houston, TX, United States

**Keywords:** fluid teams, neonatal intensive care unit, teamwork, teams, patient families, hierarchy, patient handoffs

## Abstract

Fluid teams, characterized by frequent changes in team membership, are vital in the neonatal intensive care unit (NICU) due to high patient acuity and the need for a wide range of specialized providers. However, many challenges can hinder effective teamwork in this setting. This article reviews the challenges related to fluid teamwork in the NICU and discusses recommendations from team science to address each challenge. Drawing from the current literature, this paper outlines three challenges that can hinder fluid teamwork in the NICU: incorporating patient families, managing hierarchy among team members, and facilitating effective patient handoffs. The review concludes with recommendations for managing NICU teamwork differently using strategies from team science.

## Introduction

Healthcare teams are often characterized by fluidity, or frequent changes in team membership wherein workers join or leave teams depending on the present task and his or her contribution (Bedwell et al., 2012; Mortensen and Haas, 2018; Larson and DeChurch, 2020). Fluid teams are especially critical in the neonatal intensive care unit (NICU), where patient acuity is high and many providers may be necessary to care for infants with complex medical needs during lengthy hospital stays (Derienzo et al., 2014; Masten et al., 2019; Salih and Draucker, 2019; Kaplan et al., 2020). NICU teams are multidisciplinary and bring together a wide array of medical providers, support services, and patient family members, who interact dynamically and interdependently to care for patients (Barbosa, 2013).

The purpose of this review is to highlight challenges related to fluid teamwork in the NICU and, drawing from team science, discuss opportunities to address each challenge to promote effective teamwork in the NICU. This review begins with an overview of fluid teamwork in the NICU, highlighting times when fluid teams are critical to patient outcomes. Next, the paper focuses on three challenges facing NICU teams: incorporating patient families, managing hierarchy among team members, and coordinating fluid teams during lengthy stays. Finally, the paper concludes with a discussion of avenues practitioners can use to manage NICU related teamwork. While fluid teams in the NICU face several challenges, we focus here on challenges that are (a) particularly relevant to teamwork, (b) specific to the NICU environments (or other intensive pediatric), and (c) because these challenges are associated with particularly critical outcomes. First, we focus on patient families because of their outsized impact on patient health outcomes and because patient family members are at an increased risk for mental health related issues. Support for parents’ involvement in infant care is associated with a reduction in mental health related issues (Griffin, 2006; Klawetter et al., 2022). Moreover, parental involvement, such as increased holding, lead to better developmental outcomes and a more successful and timely discharge, making it vital to examine the challenges and potential solutions to incorporating families into the NICU team (Nair et al., 2003; Pineda et al., 2018). We focus on hierarchical leadership due to its detrimental outcomes for staff morale and patient safety (Fernandopulle, 2021). Hierarchy in medical contexts is particular concern given the vulnerability of the patient. Lastly, we focus on patient handoffs due to the sheer number of patient handoffs in NICU settings, coupled with the devastating repercussions poor handoffs could have (Derienzo et al., 2014; Ediger et al., 2022).

## Fluid teams in the NICU

Although team fluidity is common across healthcare settings, it is particularly central to the care of infants in the NICU (Barbosa, 2013). Indeed, fluid teams are involved in patient care from admission to discharge and are especially vital during resuscitation, transport to the NICU, and at critical points in a patient’s care that require collaboration across medical specialties.

Fluid teams often become vital to neonatal care in the delivery room, where highly skilled providers from obstetrics and pediatrics work together to facilitate infant resuscitation. Poor teamwork is the root cause of most perinatal deaths and injury in these contexts (Thomas et al., 2007). Indeed, fluidity contributes to the challenge of delivering high quality care in these contexts. Neonatal resuscitation involves all aspects of delivery room care from initial assessment to ventilation and medication administration where necessary (Thomas et al., 2006). This context brings together providers from obstetrics and pediatrics, and for infants requiring NICU admission, an even greater number of staff may be involved. Beyond this inherent complexity, additional factors such as the involvement of medical trainees or high staff turnover within a hospital may exacerbate challenges. Effective teamwork during neonatal resuscitation can improve patient outcomes, and teams that receive training on teamwork skills demonstrate higher quality resuscitations (Thomas et al., 2010).

Transporting infants from low acuity hospitals to facilities with higher levels of care provides another context wherein teams are highly fluid (Lupton and Pendray, 2004). Transporting high risk infants requires collaboration between the neonatal transport team, which can consist of physicians, neonatal nurse practitioners, neonatal RNs, respiratory therapists, and specialized paramedics (Hansmann, 2009). This process is often unpredictable due to challenges related to staffing, the availability of ambulances or other transport equipment, and administrative support (Hansmann, 2009; Akula et al., 2020). Indeed, effective teamwork in these contexts is essential (Foronda et al., 2016). The American Academy of Pediatrics advocates for training in teamwork skills such as communication, environmental awareness, and decision; however, studies exploring the impact of improved teamwork skills on patient outcomes in transport contexts remain limited (Stroud et al., 2013; Campbell and Dadiz, 2016).

From there, teamwork in the NICU continues to be characterized by fluidity. Indeed, daily interprofessional rounds typically involve a rotating set of neonatologists, neonatal nurse practitioners, resident physicians, nurses, nutritionists, respiratory therapists, family support specialists, social workers, and staff from consulting specialties (Barbosa, 2013). The fluidity of professionals is dependent on the changing healthcare needs of the infant, therefore when the team meets during rounds to discuss the plan of care for the infant, the members present may look different throughout the course of the infant’s stay in the NICU. In addition, throughout an infant’s course of care, teams may swiftly come together to address health crises. In such cases, teams will consist of the broad array of staff whose skills are needed to treat the infant. It is critical that the team be prepared, adaptable, have open communication, and their actions be synergistic (Finer and Rich, 2002). Effective teamwork throughout a patient’s stay is essential to achieving positive patient outcomes (Thomas et al., 2004; Leggat, 2007; Masten et al., 2019).

## Challenges related to fluidity in NICU teams

While some challenges associated with fluid teamwork are common across healthcare specialties, the NICU cultivates a unique, high-stress, environment that gives rise to additional challenges and can exacerbate the difficulty and importance of these challenges (Derienzo et al., 2014; Masten et al., 2019; Salih and Draucker, 2019; Kaplan et al., 2020). As they care for critically ill infants, NICU teams are faced with myriad demands (Braithwaite, 2008). The following section focuses on three challenges unique to fluid teams in the NICU, highlighting opportunities for psychologists to help improve teamwork in this setting. These challenges and opportunities are summarized in Table 1.

**Table 1:** Challenges for fluid teams in the NICU.

Challenge	Description	Opportunities
1. Including patient families in NICU teams	Family-centered care promotes the inclusion of patient families in daily rounding (Craig et al., 2015). Various benefits to including patient family members have been seen, but it can be challenging in itself and further generates even more fluidity in teams.	NICU teams can include parents through designating tasks and activities to parents, such as reading (Jain et al., 2021). NICU teams can, and should, actively involve parents in rounds and decision making (Sigurdson et al., 2020).
2. Managing hierarchy within NICU teams	A medical hierarchy is particularly prominent in the NICU (Ediger et al., 2022). This may negatively affect psychological safety, or a shared sense that it is safe to speak up, as well as increase moral distress among nurses (Becker and Grunwald, 2000; Mills and Cortezzo, 2020).	Leaders in the NICU can combat the negative consequences of a hierarchy through utilizing an inclusive leadership style (Nembhard and Edmondson, 2006). It is imperative that those in a leadership position help cultivate psychological safety within the team (Masten et al., 2019).
3. Facilitating effective patient handoffs across teams	The vast number of patient handoffs in the NICU can put infants at risk, especially if done improperly.	Implementing formalized structure or protocol for handoffs could help lower risk of further injury or illness. Furthermore, required training could help lower mistakes and errors.

## Challenge #1: including patient families in NICU teams

Although patient families can be included in the care team across the healthcare field, the role of patient families is particularly vital in the NICU. Incorporating patient families into the care process was included as part of the National Steering Committee for Patient Safety, 2022, which detailed the importance of establishing trust between patient and practitioner and the value of engaging families in the care process. In the NICU, patient families are often vital in providing physical care for their infant, and are often solely responsible for their infant’s care at discharge. Improving family engagement in the NICU promotes positive physical, cognitive, and psychosocial development in infants that extends beyond their hospital stay (Craig et al., 2015). Family-centered developmental care advocates for parents to be included as part of the medical care team for their infants (McGrath et al., 2011a,b). However, including patient family members in the care team generates even more fluidity in teams as patient family members may not have a consistent physical presence at their infant’s bedside, typically lack medical expertise and do not have established relationships with medical staff, and are temporary members of the NICU team as even lengthy stays last months, rather than years.

First, these conditions make it difficult for medical staff to form a shared mental model with parents. Shared mental models describe a team’s shared understanding of tasks and their environment (Cannon-Bowers et al., 1993). For example, NICU teams might have a shared mental model about a baby’s current condition or a plan for their care. Patient family members without medical expertise may lack the requisite knowledge to understand their baby’s treatment and as a result, may need additional education to build a shared mental model with medically trained team members.

Second, parents’ unfamiliarity with the medical environment, lack of existing relationships with NICU staff, and emotional vulnerability during their NICU stays can make it challenging to develop trust with team members. Frequent changes in nursing staff and a lack of understanding of hospital resources lead to gaps in mutual trust, particularly among families of color and/or low socioeconomic status (Sigurdson et al., 2020). Furthermore, it is common for NICU parents to feel like providers do not understand their emotional situation, leading parents to perceive NICU staff as unempathetic, insensitive, and uninterested (Wigert et al., 2013).

## Opportunities for including patient families

Despite these challenges, team science has the potential to inform family-centered approaches to facilitate the development of shared mental models and to build trust with patient families. For example, family-centered care emphasizes the importance of including patient families in daily rounding (Craig et al., 2015). When the team is rounding on an infant, inviting parents to actively participate provides the opportunity to inform the family of plans for their baby’s care, educate families, and answer questions family members may have for the team (Griffin, 2006). Providing structure for this type of communication can provide an important foundation for building a shared mental model between patients and families. In addition, a patient’s parents prefer an active or shared approach to the decision-making process of their infant’s care (Soltys et al., 2020). Inviting family members to take part in decision making helps build trust with providers (Sigurdson et al., 2020).

Additionally, designating tasks and activities to parents has the potential to further incorporate them into the NICU team and cultivate a feeling of empowerment. For example, parents may be instructed to read to their infant (Jain et al., 2021). Reading to an infant in the NICU has been shown to increase bonding with the child, improve neurodevelopment, and promote language growth outcomes, as observed at around 2 years of age (Jain et al., 2021). Giving parents a task allows them to positively impact the development of their child, play active role within the team, and potentially improve their experience in the NICU and within the fluid team.

Neonatal nursing staff play a particularly instrumental role in including patient families in the NICU care team (Barbosa, 2013). Indeed, parents describe receiving more support from nurses than physicians or other NICU staff because nurses have the most interaction with both the infant and their family (Wigert et al., 2013). Equipping nursing staff to connect patient families to resources can help build trust between patient families and providers (Obeidat et al., 2009; Sigurdson et al., 2020). Moreover, nursing staff serve as patient families’ primary informants, and spend more time educating families on their baby’s care (Kowalski et al., 2006). As a result, nurses may be a particularly effective liaison to ensure family members can build and maintain a shared mental model with medical team members.

## Challenge #2: managing hierarchy within NICU teams

Another challenge to fluid teams’ success in the NICU is the hierarchy among medical providers. Although hierarchy is common across medical specialties, research suggests that it is particularly prominent in the NICU. In one study, nearly 92% of NICU staff agreed that a hierarchy existed within their NICU team (Ediger et al., 2022). Other work has found similarly high levels of agreement among both physicians and nursing staff, a trend which has persisted for decades (Guillemin and Holmstrom, 1986; Masten et al., 2019). Hierarchy is an important, and often beneficial, feature of medical teams. For example, in fluid teams, the inherent hierarchy present in medicine may help eliminate role ambiguity during infant resuscitation when providers must quickly and correctly intubate an infant, wherein medical residents often require multiple attempts to complete correctly (Falck et al., 2003). However, hierarchy can yield negative team outcomes when it is not properly managed, limiting team psychological safety (i.e., the shared sense that it is safe to speak up), leading nurses to act on physician orders even when they disagree with the decision based on their knowledge and experience with an infant (Becker and Grunwald, 2000; Nembhard and Edmondson, 2006).

Notably, patient families can also be negatively impacted by hierarchy in NICU teams. The characteristics of NICU parents, who often lack medical expertise and knowledge of the healthcare system, often place parents near the bottom of a hierarchy regarding their child’s care (Hurst, 2001; Sigurdson et al., 2020; Harrison et al., 2021). However, at other times, parents may have final say in medical decisions on behalf of their child, with the right to refuse or discontinue treatment (McNary, 2014; Gibson, 2022). The way the medical team describes infants’ status or next steps for patient care can warp parental autonomy (Orfali, 2004). In fact, parents have been described as second-order patients, and attending physicians are particularly likely to give less weight to parental issues (Becker and Grunwald, 2000). This contrasts against the reported need to involve parents in decision making (Harrison, 1993; Soltys et al., 2020).

## Opportunities for managing hierarchy

Managing hierarchy in the NICU by taking steps to build psychological safety can help create a work environment that allows team members to have open communication with each other and safely voice their opinions and disagree with each other (Masten et al., 2019). Doing so can improve fluid teams’ collaboration, ensuring members quickly develop a shared sense that it is safe to speak up without fear of retribution (Nembhard and Edmondson, 2006). As is the case across medical teams, an attending physician or other designated leader’s leadership style has outsized influence on the communication, trust, job satisfaction, and performance among NICU staff (Nanjundeswaraswamy and Swamy, 2014; Chatterjee et al., 2018).

Inclusive leadership can help combat the negative effects of position hierarchy in the NICU and has been shown to improve psychological safety (Nembhard and Edmondson, 2006). While leaders may be assigned to work with different teams, those who hold a leadership position in the NICU (e.g., attending physicians), are relatively consistent, making developing excellent physician leaders beneficial. Indeed, leaders are arguably the most effective facilitator for building psychological safety among the team, which is imperative for team function in the NICU (Nembhard and Edmondson, 2006; Torralba et al., 2020). One way to aid leadership development in this area could be to train leaders who rotate among teams, which has proven beneficial in other healthcare settings (Blumenthal et al., 2012; Sonnino, 2016).

## Challenge #3: facilitating effective patient handoffs across teams

The nature of infants’ arrival to the NICU and complicated, lengthy hospital stays necessitates coordination between fluid teams, as well as within fluid teams. One prominent manifestation of this challenge is patient handoffs, which are incredibly common for NICU teams. A unique challenge that arises for fluid teams in the NICU is the number of patient handoffs (Ediger et al., 2022). For example, an infant in the NICU for roughly 6 months will experience around 300 nurse handoffs alone (Gray et al., 2010). Poorly managed patient handoffs can result in dire consequences, and critically ill patients are at an even higher risk of communication-related errors during handoffs (Wohlauer et al., 2012; Derienzo et al., 2014). Handoffs in the NICU also require consideration of the patient’s family, whose presence is variable and not guaranteed at the time of a handoff. The patient handoffs require communication between multiple fluid teams, making the process susceptible to risk. While nurse–nurse handoffs are most frequent, handoffs from labor and delivery occur at the start of an infant’s stay in the NICU which is followed by multiple fluid team members from physicians, respiratory therapists, fellows, etc. contributing to the rate of patient handovers (Gephart, 2012; France et al., 2019; Cardona et al., 2021). A potential complication to patient handoffs is that the shared mental model between the fluid team during the day may not be transferred over into the night shift, and vice versa. Shared mental models can promote better communication and increase the team’s overall success (Edgar et al., 2021), but if a shared mental model is lost during shift transfer, it could act as a barrier to successful teamwork (Kilcullen et al., 2022).

## Opportunities for managing patient handoffs

In other medical contexts, best practices for conducting handoffs typically include using formalized structures or protocols for handoffs (Wolf et al., 2023). This approach may prove particularly useful for fluid teams, where relying on a protocol ensures that the most important information is transmitted between teams no matter who is present. Formalized training on handoffs or debriefs can be a particularly effective avenue for improving patient handoffs (Salih and Draucker, 2019; Wolf et al., 2023). It is worth noting that due to the interdisciplinary nature of a fluid team in the NICU, the medical staff could have different variations in handoff education and training, resulting in differing quality in patient debriefs (Cardona et al., 2021). Formalized training for all NICU staff has the potential to combat some of the communication errors and risks when it comes to patient handoffs.

## Discussion: managing NICU teamwork differently

This review highlights three challenges and opportunities facing fluid teams in the NICU; however, beyond these discrete issues, there are many overarching approaches to redesign NICU teamwork to enhance effectiveness (Table 2).

**Table 2:** Practical recommendations for teams in the NICU.

Strategies for managing NICU teamwork differently	Recommendations for implementation
1. Team training	Tailored trainings offer the opportunity for practitioners to continually improve patient handoffs, and both individual and teamwork skills (i.e., communication and decision making). Psychological safety can also be improved through team training as well as shared mental models (Kilcullen et al., 2022). Actively training medical staff based on needs can have an immense impact on improving teamwork (Kilcullen et al., 2022). Including patient families in teamwork skills training could encourage more effective collaboration with patient families
2. Team debriefing	Due to the unique nature of the NICU, increased communication remains vital. One method that practitioners can implement to increase communication, improve teamwork, and ensure information is being shared consistently and accurately, is through multiple debriefs and within the fluid teams (Salih and Draucker, 2019). Structuring multiple debriefs and/or huddles throughout shifts could aid in communication errors and continue to establish shared mental models across team members.
3. Inclusive leadership	Inclusive leadership has the ability to navigate the hierarchical nature of the NICU as well as influence team culture and performance (Nembhard and Edmondson, 2006). Practitioners can routinely evaluate unit leaders through leadership assessment measures and/or safety culture assessments (Kilcullen et al., 2022). A further recommendation would be for leaders to leadership trainings both prior to beginning their leadership role as well as routinely throughout.

**Recommendation 1:** *Implement team development interventions tailored to address challenges specific to fluid NICU teams.*

Team development interventions describe intentional efforts and interventions used to improve team performance, such as team training, team debriefing, and team leadership (Shuffler et al., 2018). These team development interventions should be tailored to account for fluidity and the unique challenges facing NICU teams, and might benefit from additional focus on building individual teamwork competencies that could be transported into any team setting (Cannon-Bowers et al., 1995). For example, team training is a proven method for improving teamwork in healthcare contexts broadly (Hughes et al., 2016). However, fluid teams require a tailored approach focused on the aspects of teamwork most likely to see challenges due to their fluidity. For example, NICU teams must be trained on how to develop shared mental models quickly to respond to rapid changes in team membership. Training tailored to fluid NICU teams could offer the opportunity for practitioners to continually improve patient handoffs, or quickly building team psychological safety or trust (Kilcullen et al., 2022). Given the rapid changes in membership inherent in fluid teams, structured and heavily routinized team development interventions may also prove more effective. For example, requiring providers to use a checklist during patient handoffs provides consistency in shared information despite the instability in which team members may be present during a handoff.

Due to their fluidity, NICU teams may also benefit form training or other interventions focused on individual, rather than team, behavior. Individuals should be trained on both individual and team level skills, such as coordination, backup-behavior, information sharing, shared leadership, and other generalizable teamwork skills (Bedwell et al., 2012). Such training ensures that individual team members possess a common set of teamwork skills that can be applied with any team. Further, organizational support plays an important role in influencing individual motivation to participate in in training and transferring behaviors learned in training to the work environment, and thus should also be prioritized (Salas et al., 2018).

Team training is a proven method for improving team performance across a wide range of settings, including in healthcare (Hughes et al., 2016). Further, training on the knowledge, skills, and abilities detailed above, such as building shared mental models and facilitating coordination, have received robust support. However, research on the outcomes of such training in fluid teams in particular is scant. Future research might examine how team training and teamwork skills training impact performance in fluid teams.

**Recommendation 2:** *Train NICU families to help them become effective team members.*

Although patient family members often receive informal training from NICU staff regarding their infant’s care, patient family members may also benefit from tailored trainings targeting communication skills, advocacy, as well as more formalized education on practical skills to help with the care of their infant, such as good hygiene practices or the importance that their presence and talking to the infant can have. Although we could not identify any research on such an approach to training, it may be an important avenue for future work exploring patient families as team members in the NICU. For example, patient families may benefit from training on psychological safety, which may encourage them to speak up or recognize when a medical provider could do a better job promoting psychological safety. Patient families may benefit from training similar to that provided to medical team members surrounding building shared mental models or information sharing practices, such as using closed loop communication to ensure information accuracy. Although similar training has proven effective in healthcare contexts (Hughes et al., 2016), to our knowledge, no research has examined the impact of training patient family members on teamwork skills. Thus, this is a potentially important avenue for future research.

**Recommendation 3:** *Structuring team debriefs and huddles and tailoring these interventions to the NICU environment could further promote effectiveness.*

To ensure information is being shared consistently and accurately across teams and shifts, structuring and/or requiring debriefs at critical points in time (e.g., at shift change) could aid in addressing communication errors and continue to establish shared mental models across team members. Due to the constant fluctuation of team members, establishing a standard protocol for debriefs could ensure more fruitful debriefs when individuals transfer across teams. Although checklists for patient handoffs and huddles are common in subdisciplines such as surgery, such approaches are newer to NICU environments (Manzo et al., 2023). Handoffs protocols could be developed systematically, with input from all providers who might be involved in common handoffs, such as from the delivery room to NICU or as part of neonatal transport, to ensure all relevant information is shared. Similarly, rounding or other types of huddles could be structured to ensure NICU teams discuss any issues relevant to patient care as well as plans for communicating with patient family members after a round. Given the success of structured tools, such as debriefs and handoffs, across a wide variety of other contexts including in healthcare, these tools can be implemented in NICU teams with confidence (Keiser and Arthur, 2021; Manzo et al., 2023). However, additional research to capture the impact of these tools in the NICU setting specifically would be beneficial.

**Recommendation 4:** *Tailor leadership training to address the challenges facing NICU teams.*

Inclusive leadership can combat the hierarchical nature of the NICU as well as influence team culture and performance (Nembhard and Edmondson, 2006; Kilcullen et al., 2022). Redesigning leadership training could help develop inclusive leadership skills in attending physicians. For example, leaders should be trained on how to build psychological safety both among NICU staff and with patient families. Given the outsized role leaders play in shaping their teams, it is essential that leaders are particularly effective at the team-based skills discussed across our recommendations. Although leadership training has received robust empirical support across many contexts (Lacerenza et al., 2017), training focused on inclusive leadership has received limited empirical attention. Accordingly, this is a fruitful avenue for future research both in and outside of the NICU.

Fluid teams are critical throughout an infant’s time in the NICU, including during delivery, transport, and in the day-to-day care of infants (Finer and Rich, 2002; Hansmann, 2009; Barbosa, 2013). However, NICU teams face challenges to effective fluid teamwork (Masten et al., 2019; Soltys et al., 2020; Ediger et al., 2022). This paper details these challenges, noting opportunities for recommendations from team science that may improve teamwork in the NICU. Addressing these challenges to successful teamwork would be an enormous step in improving both the safety of patients in the NICU, as well as potentially impacting job satisfaction and psychological safety among the team (Nembhard and Edmondson, 2006; Braithwaite, 2008).

## Author contributions

EB: Writing – original draft, Writing – review & editing. GR: Writing – original draft, Writing – review & editing. AT: Conceptualization, Funding acquisition, Writing – original draft, Writing – review & editing. BO: Funding acquisition, Writing – review & editing. ES: Conceptualization, Funding acquisition, Supervision, Writing – review & editing.
